# Enhancement of Electrocatalytic and Pseudocapacitive Properties as a Function of Structural Order in A_2_Fe_2_O_5_ (A = Sr, Ba)

**DOI:** 10.3390/molecules28165947

**Published:** 2023-08-08

**Authors:** Surendra B. Karki, Farshid Ramezanipour

**Affiliations:** Department of Chemistry, University of Louisville, Louisville, KY 40292, USA

**Keywords:** structural order, electrocatalysts, oxygen evolution reaction, hydrogen evolution reaction, pseudocapacitance

## Abstract

Significant enhancements of electrocatalytic activities for both half-reactions of water-electrolysis, i.e., oxygen evolution reaction (OER) and hydrogen evolution reaction (HER), as well as pseudocapacitive charge-storage properties are demonstrated upon changing the structural order in a perovskite-type system. The structural change is prompted by the increase in the ionic radius of the A-site ion in A_2_Fe_2_O_5_. The structure of Sr_2_Fe_2_O_5_ consists of alternating layers of FeO_6_ octahedra and FeO_4_ tetrahedra, whereas Ba_2_Fe_2_O_5_ comprises seven different coordination geometries for Fe. We note that the catalytically active metal, i.e., iron, and the oxygen stoichiometry are the same for both materials. Nevertheless, the change in the structural order results in significantly greater electrocatalytic activity of Ba_2_Fe_2_O_5_, manifested in smaller overpotentials, smaller charge-transfer resistance, greater electrocatalytic current, and faster reaction kinetics. In addition, this material shows significantly enhanced pseudocapacitive properties, with greater specific capacitance and energy density compared to Sr_2_Fe_2_O_5_. These findings indicate the important role of structural order in directing the electrochemical properties.

## 1. Introduction

Efficient electrochemical energy conversion and storage devices require the development of functional materials with enhanced properties. Oxide materials have shown great promise for various applications, such as in water electrolyzers, solid oxide fuel cells (SOFCs), oxygen separation membranes, and oxygen sensors. Oxide systems derived from the perovskite structure are of particular interest, due to their interesting properties [1,2,3]. They exhibit a number of structures, which can be achieved by different methods, such as the partial removal of oxygen, which can be used to promote oxygen diffusivity and surface exchange kinetics [4,5].

Transition metal oxides adopting the perovskite structure have the general formula ABO_3_ (A = alkali, alkaline earth or lanthanide, B = usually transition metal). Oxygen-deficient perovskites (ODPs) can also be prepared, when some of the oxygen atoms are lost, giving the formula ABO_3-δ_, where δ is the number of oxygen vacancies per unit formula. Oxygen defects in such structures may be spread randomly or order systematically to yield several possible structures. ODPs having the oxygen deficiency of δ = 0.5, i.e., ABO_2.5_ or A_2_B_2_O_5_, often form the brownmillerite structure. Brownmillerites have been studied for a number of applications, such as oxygen carriers (Ca_2_Fe_2_O_5_) [6], photochemical oxygen production (Sr_2_Fe_2_O_5_) [7], oxygen ion and proton conduction (Ba_2_In_2_O_5_) [8], supercapacitors (Ca_2_FeCoO_5_) [9], and oxygen evolution (Ca_2_FeCoO_5_) [10].

Among these technologies, electrocatalytic water splitting is of great interest [11,12,13]. Water splitting has two half reactions, namely, hydrogen evolution reaction (HER) i.e., 2H_2_O + 2e^−^ → H_2_ + 2OH^−^, and oxygen evolution reaction (OER) i.e., 4OH^−^ → O_2_ + 2H_2_O + 4e^−^, in alkaline medium. However, both reactions have sluggish kinetics, leading to large overpotentials. The overpotentials can be significantly reduced by electrocatalysts. Traditional benchmark catalysts for HER and OER have been those based on precious metals. For example, Pt is a benchmark electrocatalyst for HER, which shows the best performance in both acidic and alkaline media [14]. Materials that have HER electrocatalytic activities comparable to Pt are scarce. Some of the traditional benchmark electrocatalysts for OER include RuO_2_ [15] and IrO_2_ [16]. While these catalysts show high activities for OER catalysis, the cost and scarcity of noble metals is a significant problem. Therefore, alternative materials have been explored to reduce the cost and make the water-splitting process more practical [17]. Perovskite oxide-based catalysts have emerged as promising alternatives, particularly for OER [18], Along with the use of earth abundant metals, it is highly desired to have stable catalysts that can operate under electrolysis conditions for extended periods of time. Perovskite oxides have shown high stability, especially in alkaline medium. These oxides can also have a variety of crystal structures. Many different metals from the periodic table can be used to modify the compositions and electrocatalytic properties to achieve efficient water splitting. Different approaches have been examined in an effort to enhance the electrocatalytic activity of perovskite oxides by metal substitutions in A or B-sites. For instance, we have previously explored the Ca substitution in the perovskite La_1/3_Sr_2/3_FeO_3-δ_ to obtain a bilayer brownmillerite phase, La_1/3_Ca_2/3_FeO_3-δ_, with enhanced OER and HER activities [19]. Another example is the systematic substitution of Ca into the perovskite SrFe_1/2_Co_1/2_O_3-δ_, leading to the ordering of oxygen-vacancies in CaFe_1/2_Co_1/2_O_3-δ_, which has a brownmillerite structure and shows significantly lower OER and HER overpotentials [10]. Other examples, such as the changes in OER activity by varying the degree of Sr substitution in La_1−x_Sr_x_NiO_3_, have also been investigated [20].

Another electrochemical property that is exhibited by oxide materials, particularly perovskite oxides, is pseudocapacitive charge storage. Pseudocapacitors behave somewhat similar to traditional capacitors but also include electron transfer reactions [21,22]. Their charge storage process involves both the formation of electric double-layer charge separation and reversible surface redox (faradic) reactions [22]. There are various types of pseudocapacitance, namely, redox pseudocapacitance, underpotential deposition, and intercalation pseudocapacitance [23]. In redox pseudocapacitance, both electrochemical adsorption of ions and faradaic charge-transfer take place on or near the surface of a material. Underpotential deposition is another mechanism, which occurs by the formation of a monolayer of metal ions above their redox potential on the surface of a different metal. Intercalation pseudocapacitance, which is the focus of this study, is another phenomenon that involves ion intercalation and a faradaic charge-transfer. This process has been observed in perovskite oxide materials. An example is the perovskite LaMnO_3_ [24], where a reversible intercalation of the oxide anion into the material takes place while Mn ions undergo a reversible redox reaction [24]. The mechanism involves several steps, initiated by the adsorption of hydroxide ion, which then loses a proton to leave behind an oxide ion that is intercalated into the material. As with many oxide properties, pseudocapacitance is also affected by structural changes. We have previously shown an example of the enhancement of pseudocapacitive properties by changing the structure from a disordered oxygen-deficient perovskite in SrCa_2_GaMn_2_O_8_ to an ordered brownmillerites system in Ca_3_GaMn_2_O_8_ [25]. Another example is the improvement of pseudocapacitive properties upon partial substitution of lanthanum by potassium in LaFeO_3_ to form La_0.5_K_0.5_FeO_3_ [26]. Similarly, the effects of Ca or Sr doping at the A-site of LaMnO_3_ on the pseudocapacitive properties have been investigated [27,28].

In the present work, we demonstrate considerable enhancements in electrochemical properties upon structural changes prompted by the change in the ionic radius of the A-site ion in A_2_Fe_2_O_5_ (B = Sr^2+^, Ba^2+^). Some properties of these materials, such as electrical conductivity and magnetism, have been studied before [29,30,31]. In this work, we investigate the electrochemical properties for water-electrolysis and pseudocapacitive energy storage. While both materials are synthesized under identical conditions and contain the same oxygen stoichiometry, the change in the structural order leads to a significant improvement of the electrochemical properties of Ba_2_Fe_2_O_5_ over Sr_2_Fe_2_O_5_. The former shows enhanced electrocatalytic activities for both half-reactions of water electrolysis, OER and HER, as well as significantly greater pseudocapacitive charge storage properties.

## 2. Results and Discussion

### 2.1. Crystal Structure

**Figure 1:**
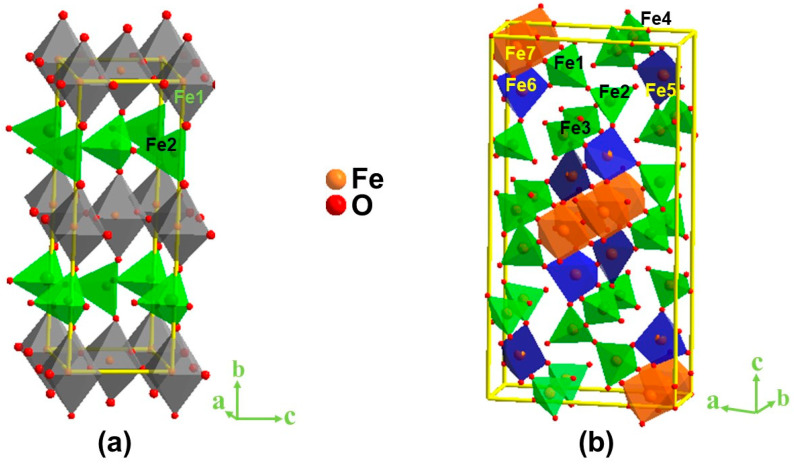
Crystal structures of (**a**) Sr_2_Fe_2_O_5_ and (**b**) Ba_2_Fe_2_O_5_. The former has two crystallographically distinct Fe sites, while the latter has seven distinct Fe sites. The A-site atoms residing in spaces between the above polyhedra are omitted for clarity.

Crystal structures of both compounds were confirmed by Rietveld refinement analyses (Figure S1 and Tables S1 and S2) using powder X-ray diffraction data. The crystal structures of both materials were consistent with previous reports [30,31,32]. We note that both materials were synthesized under identical conditions in an argon atmosphere using the same iron precursor. Iodometric titrations were used to quantify the oxygen content, showing five oxygens per formula unit for both compounds. Therefore, the variation in their crystal structure is related to the change in the ionic radius of the A-site metal from 1.44 Å for Sr^2+^ to 1.61 Å for Ba^2+^ [33], leading to a significant change in the structural order. Sr_2_Fe_2_O_5_ has a brownmillerite structure [29,30], containing two distinct Fe positions, one with octahedral and another tetrahedral coordination environment. This leads to the formation of alternating layers of FeO_6_ octahedra and FeO_4_ tetrahedra in Sr_2_Fe_2_O_5_ (Figure 1a). The Sr^2+^ ions reside in spaces between these layers. On the other hand, the larger ionic radius of Ba^2+^ results in a significantly more complex structure for Ba_2_Fe_2_O_5_ [30,31]. This material contains seven different Fe positions, with several different coordination geometries, which may be described as tetrahedral, square pyramidal, and octahedral, although some polyhedra are significantly distorted, as shown in Figure 1b. The change in the structural arrangement between the two materials leads to major variations in electrochemical properties as described in the next sections.

### 2.2. Electrocatalytic Properties for OER and HER

The electrocatalytic activities of both compounds toward both half-reactions of water-splitting, namely, oxygen evolution reaction (OER) and hydrogen evolution reaction (HER) were studied in alkaline conditions.

Several parameters are often utilized to compare the activities of different electrocatalysts. One parameter is the onset potential, which refers to the start of the faradaic process, marked by a rise in the current density. Another parameter for gauging the catalytic performance is the overpotential beyond the ideal thermodynamic potential at a current density of 10 mA/cm^2^ (η_10_), which is associated with 10% solar-to-fuel conversion efficiency of a device in solar fuel synthesis [34]. Catalysts that enable the OER and HER at lower onset and overpotential are desired, as they lower the energy required for these processes to occur [35].

**Figure 2:**
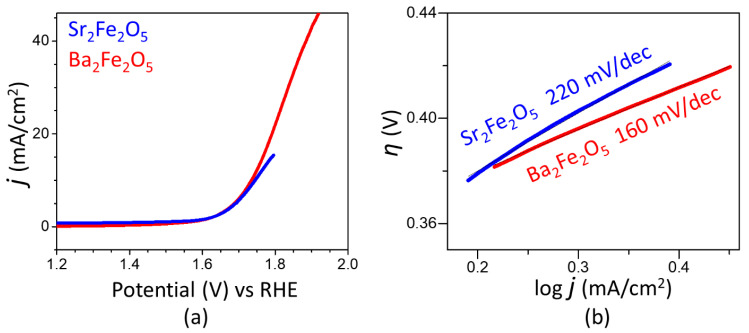
(**a**) OER polarization curves in 1 M KOH. (**b**) Tafel plot showing Tafel slopes for both compounds.

The electrocatalytic activities of the two materials for OER are represented by the polarization curves in Figure 2a. As observed in this plot, the two compounds show a similar onset potential at about 1.6 V. The overpotential for OER is calculated as η_10_ = E_RHE_ − 1.23 V, where E_RHE_ is the potential versus RHE at 10 mA/cm^2^, and 1.23 V is the thermodynamic potential for OER. The overpotential for Ba_2_Fe_2_O_5_ (0.50 V) is slightly lower than that of Sr_2_Fe_2_O_5_ (0.52 V). Importantly, the utilization of Ba_2_Fe_2_O_5_ as a catalyst leads to a current response, which is several-folds greater than that obtained using Sr_2_Fe_2_O_5_. This indicates the significantly higher electrocatalytic activity of Ba_2_Fe_2_O_5_ compared to Sr_2_Fe_2_O_5_. The OER activity of Ba_2_Fe_2_O_5_ is retained for many hours, as shown in the chronopotentiometry data in the inset of Figure 2a, indicating the stability of this catalyst. The kinetics of electrochemical reactions can be evaluated using the Tafel equation, η = a + b log *j*, where *η* is overpotential and *j* is current density. The linear fit of the plot of η versus log *j*, using the overpotential from the curved region of the polarization curve, will give the Tafel slope [25,36,37,38,39]. Faster reaction kinetics is associated with a smaller Tafel slope, indicating a more facile electron transfer. As shown in Figure 2b, the Tafel slope for Ba_2_Fe_2_O_5_ is smaller than that of Sr_2_Fe_2_O_5_, indicating a faster OER process, which is consistent with the higher electrocatalytic activity of Ba_2_Fe_2_O_5_.

**Figure 3:**
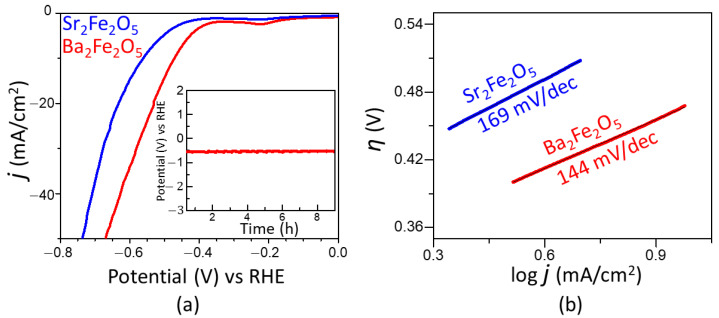
(**a**) HER polarization curves in 1 M KOH. The inset shows chronopotentiometry data of Ba_2_Fe_2_O_5_ at a current density of −10 mA/cm^2^. (**b**) Tafel plot showing Tafel slopes for both compounds.

**Figure 4:**
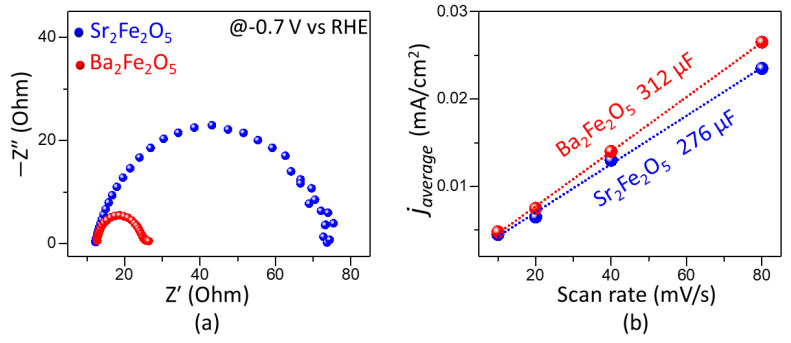
(**a**) Nyquist plot for electrochemical impedance spectroscopy data obtained in the HER potential region of −0.7 V vs. RHE. (**b**) Plots of j_average_ vs. scan rate obtained from CVs in the non-faradaic region, giving the C_dl_ values as slope.

The electrocatalytic activities of the two materials for HER are represented by the polarization curves in Figure 3a. As observed in this plot, the onset potential obtained using Ba_2_Fe_2_O_5_ (−0.35 V) is lower than that of Sr_2_Fe_2_O_5_ (−0.40 V). The overpotential for HER is calculated as η_10_ = E_RHE_ − 0.0 V, where E_RHE_ is the potential versus RHE at 10 mA/cm^2^, and 0.0 V is the thermodynamic potential for HER. The overpotential obtained for Ba_2_Fe_2_O_5_ (−0.47 V) is considerably lower than that of Sr_2_Fe_2_O_5_ (−0.56 V). We note that the overpotential for the precious metal benchmark catalyst Pt/C (20 wt. % Pt) has been reported to be close to −0.02 V vs. RHE [40]. However, the overpotential of Ba_2_Fe_2_O_5_ is lower than those of some other reported oxide catalysts, such as Sr_3_Mn_2_O_6_ (−0.59 V) [41] and SrLaCoO_4-δ_ (−0.541 V) [36] and SrLaFeO_4_ (−0.691 V) [36]. The HER activity of Ba_2_Fe_2_O_5_ is retained for at least 15 h, as shown in the chronopotentiometry data in the inset of Figure 3a, indicating the stability of this catalyst. In addition, as shown in Figure 3b, Ba_2_Fe_2_O_5_ results in a smaller Tafel slope compared to Sr_2_Fe_2_O_5_, indicating the faster kinetics of HER process enabled by Ba_2_Fe_2_O_5_, consistent with its greater electrocatalytic activity. This is consistent with the electrochemical impedance spectroscopy data in the HER region (Figure 4a), which shows a smaller charge-transfer resistance for Ba_2_Fe_2_O_5_, indicating a more facile electron transfer compared to Sr_2_Fe_2_O_5_.

We have also evaluated the double-layer capacitance, C_dl_, in the non-faradic region [42], where the current is generated mainly from electrical double layer charge and discharge, without contributions from electrode reactions [42,43]. The importance of C_dl_ is that it is directly related to the electrochemically active surface area [42,44,45,46]. The value of C_dl_ is determined from the equation C_dl_ = j_average_/ν [47,48], where j_average_ is the average of the absolute values of the anodic and cathodic current-densities at the middle potential of the CVs in a non-faradic region (Figure S2). The slope of the plot of j_average_ vs. scan rate gives the C_dl_. As shown in Figure 4b, C_dl_ was calculated from CVs at scan rates of 10, 20, 40, and 80 mV/s. Ba_2_Fe_2_O_5_ shows a greater C_dl_ value, which is consistent with its higher electrocatalytic activity compared to Sr_2_Fe_2_O_5_.

### 2.3. Pseudocapacitive Charge-Storage Properties

The important effect of structural changes on electrochemical properties is further demonstrated by investigation of the pseudocapacitive energy storage in the two materials. Pseudocapacitors store energy based on the faradaic processes that occur at or near the surface. Therefore, their properties lie in between those of traditional capacitors and batteries. Thus, in theory, they should be able to deliver both reasonable energy-density and power-density [49]. Pseudocapacitive properties in some oxides have been observed to occur by a reversible intercalation of the oxide anion [24,50,51]. As described by other researchers before [24,50], the process begins with the adsorption of the hydroxide ion on the electrode surface, followed by a loss of proton to another hydroxide ion to produce water and leave behind an oxide anion, which is intercalated into the electrode material [24,50].

**Figure 5:**
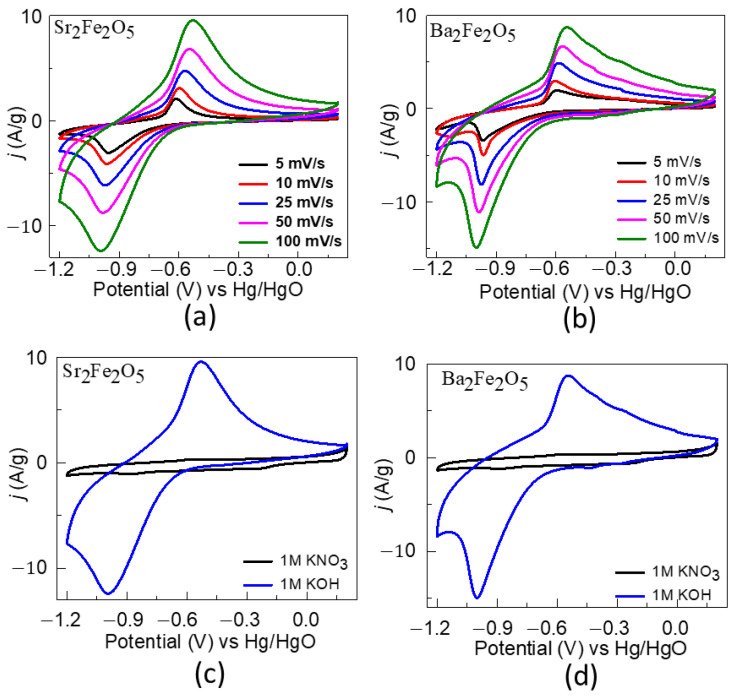
(**a**,**b**) show CVs obtained using a three-electrode setup in 1 M KOH. (**c**,**d**) show comparisons of CVs obtained in 1 M KOH (blue) and 1 M KNO_3_ (black) electrolytes.

The pseudocapacitive properties for the two materials were studied using the cyclic voltammetry data in a three-electrode cell configuration at scan rates of 5, 10, 25, 50, and 100 mV/s, as shown in Figure 5. The redox peaks are indicative of the faradaic reactions [24,52,53,54]. The redox peaks correspond to the Fe^2+/3+^ redox behavior for both compounds [52]. As observed in the CVs, as the scan rate increases, the oxidation peak shifts toward higher potentials and the reduction peak toward lower potentials. Such shifts occur due to the internal resistance of the electrode [55,56]. Moreover, the higher intensities of the redox peaks at higher scan rates are ascribed to the fast electronic and ionic transports [50,57]. Importantly, the redox peaks are not observed in a KNO_3_ solution (Figure 5), confirming that the faradaic processes arise from the oxide ion intercalation facilitated by OH^-^ in an alkaline electrolyte [52].

**Figure 6:**
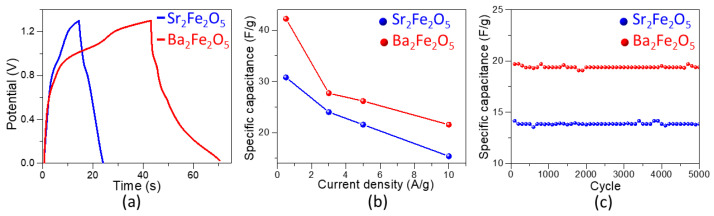
(**a**) Galvanostatic charge–discharge (GCD) profiles at 0.5 A/g for symmetric cells of each material. (**b**) Secific capacitance from GCD of symmetric cells at current densities of 0.5, 3, 5, and 10 A/g. (**c**) Stability test over 5000 GCD cycles.

A symmetric two-electrode cell was constructed by loading the catalyst ink on two Ni foam electrodes with the area of 1 cm^2^. The galvanostatic charge–discharge (GCD) was then studied using this cell in the potential window of 0.0 to 1.3 V. The shape of the GCD cycle profiles at 0.5 A/g, shown in Figure 6a, is typical of pseudocapacitors [27,28]. The specific capacitance, *C_s_*, of a two electrode cell is obtained using the equation [58,59]:

$$C_{s}=\frac{4I∆t}{m∆V}$$

In this equation, *I* is the constant applied current, ∆V is the potential window, ∆t is the discharge time, and m is the total mass of the material loaded on both electrodes. The GCD experiments were done at various current densities, 0.5, 3, 5 and 10 A/g. The *C_s_* values at each current density are shown in Figure 6b. As commonly observed in pseudocapacitors, the *C_s_* decreases with the increase in current density [27,28]. At the current density of 0.5 A/g, the *C_s_* values for Sr_2_Fe_2_O_5_ and Ba_2_Fe_2_O_5_ are ~31 F/g and ~42 F/g, respectively, indicating the significantly higher pseudocapacitive properties of the latter material. The specific capacitance from the symmetric cell of Ba_2_Fe_2_O_5_ is also superior to those of several previously reported oxide pseudocapacitors, such as La_0.5_Ca_0.5_MnO_3_ [27] and La_0.85_Sr_0.15_MnO_3_ [28], which show the respective specific capacitance values of ~6.5 F/g and less than ~8 F/g for symmetric cells at 0.5 A/g.

The energy density of the two-electrode cell is calculated by the following equation [60]:

$$E=\frac{C_{s}V^{2}}{2\times 3.6}$$

In this equation, *C_s_* is the specific capacitance from the two-electrode cell and *V* is the potential window in the GCD cycle. The constant 1/3.6 leads to the energy density in the unit of Wh/kg, considering that 1W = 1V × 1A and 1F = 1$\frac{A\cdot s}{V}$. This energy density (*E*) value is further utilized to obtain the power density using the following equation [60]:

$$P=\frac{E\times 3600}{∆t}$$

In this equation, ∆t is the discharge time in seconds and 3600 is a multiplier used to express the power density in W/kg. The two materials, Sr_2_Fe_2_O_5_ and Ba_2_Fe_2_O_5_, can deliver energy densities of ~7 Wh/kg and ~10 Wh/kg, respectively, at a power density of 1300 W/kg from a symmetric cell at a current density of 0.5 A/g. Therefore, the symmetric cell of Ba_2_Fe_2_O_5_ shows a significantly greater energy density, which outperforms some of the previously reported pseudocapacitors. An example is La_0.85_Sr_0.15_MnO_3_, where the energy density can be calculated as ~1.6 Wh/kg at 0.5 A/g, based on a specific capacitance of ~8 F/g from a symmetric cell with a reported potential window of 1.2 V [28]. Since the GCD discharge time at 0.5 A/g is not reported for the symmetric cell of La_0.85_Sr_0.15_MnO_3_, the power density cannot be estimated at 0.5 A/g. However, a higher energy density of 3.9 Wh/kg is reported for a low power density of 120 W/kg for the symmetric cell of La_0.85_Sr_0.15_MnO_3_ [28]. Similarly, the symmetric cell of Ba_2_Fe_2_O_5_ shows a better performance than that of La_0.5_Ca_0.5_MnO_3_ [27], where an energy density of 1.3 Wh/kg can be calculated based on the specific capacitance of ~6.5 F/g at 0.5 A/g for a symmetric cell with a reported potential window of 1.2 V [27]. For La_0.5_Ca_0.5_MnO_3_, a higher energy density of 7.6 Wh/kg is reported at a low power density of 160 W/kg [27].

Finally, stability studies for 5000 cycles for both materials were done using the two-electrode symmetric cell at a current density of 10 A/g. As shown in Figure 6c, both materials are stable and maintain a nearly constant specific capacitance even after 5000 cycles.

## 3. Experimental Methods

Both materials, Sr_2_Fe_2_O_5_ and Ba_2_Fe_2_O_5_, were synthesized by solid state synthesis method. The powders of the precursor compounds BaCO_3_, SrCO_3_, and Fe_2_O_3_ were ground and mixed thoroughly using agate mortar and pestle, pressed into pellets, and heated in argon at 1200 °C for 48 h (with an intermediate grinding and pelletizing). The phase purity and structures of the polycrystalline samples were confirmed by powder X-ray diffraction (XRD) at room temperature using Cu Kα1 radiation (λ = 1.54056 Å). The XRD data were analyzed by Rietveld refinement using the GSAS software [61] with EXPEGUI interface [62]. Iodometric titrations were performed by dissolving about 50 mg of the sample and excess KI (~2 g) in 100 mL of argon-purged 1 M HCl. Then, 5 mL of this solution was titrated against 0.025 M Na_2_S_2_O_3_, where 0.2 mL of a starch solution was added near the end point of the titration to act as the indicator. All iodometric titrations were performed under an argon atmosphere.

Electrocatalytic activities were measured using a three-electrode electrochemical workstation. A glassy carbon electrode coated with the catalyst ink, a commercial platinum electrode (for OER), or graphite rod (for HER) and Hg/HgO (in 1 M NaOH) were used as working, counter, and reference electrodes, respectively. The working electrode was prepared by drop-cast method for which the catalyst ink was prepared as described in our previous work [19,63], by mixing 35 mg of the catalytic material with 40 µL nafion, 7 mg carbon black, and 7 mL tetrahydrofuran (THF), followed by sonication for 15 min. The ink (40 μL) was loaded onto a glassy carbon electrode with a diameter of 5 mm and area of 0.196 cm^2^. Before starting each measurement, the 1 M KOH electrolyte (prepared in 18 MΩ nano pure water) was bubbled with argon gas for at least 30 min. Solution resistance values of ~10–22 Ω were recorded using electrochemical impedance spectroscopy in 0.1–100 kHz. All OER/HER potentials were iR-corrected. The potential can be converted to potential vs. the reversible hydrogen electrode (RHE) according to the Nernst equation [64], E_RHE_ = E_Hg/HgO_ + 0.059 pH + E⁰_Hg/HgO_, where E⁰_Hg/HgO_ = 0.098 V. In this work, the conversion to RHE potential was also verified by electrode calibration in the 1 M KOH. As shown in the Supporting Information, the Hg/HgO reference electrode was calibrated using Pt wires as the working and counter electrodes to run a cyclic voltammogram at a scan rate of 1 mV/s, and the average of the forward and return scans where the current crossed zero was taken as the thermodynamic potential [65]. This potential value (0.923 V) was nearly identical to that expected for 1 M KOH (pH = 14), i.e., 0.924 V. Therefore, this potential could be directly added to the experimental values to convert them into potentials vs. RHE [66]. Chronopotentiometry at 10 mA/cm^2^ for HER was performed by loading 40 µL of the ink on to glassy carbon electrode.

Pseudocapacitive properties were studied using both two- and three-electrode systems. For a two-electrode system, a symmetric cell was fabricated as described in the literature [67] to investigate the pseudocapacitive properties by galvanostatic charge–discharge (GCD) studies. The cell consisted of two Ni foams, separated by glass fiber filter paper, and sandwiched between two gold leads, connected to gold wires. In addition, 100 μL of the oxide ink was drop-casted on each electrode at 20 μL increments to obtain a total mass loading of ~1 mg/cm^2^. The electrodes were air-dried overnight. For three-electrode cells for pseudocapacitive experiments, the working electrode was prepared by drop-casting 10 µL of the oxide ink on a glass carbon electrode and overnight drying. CVs were run at scan rates of 5, 10, 25, 50, and 100 mV/s using a rotating disc electrode setup. All pseudocapacitive potentials are reported vs. Hg/HgO.

## 4. Conclusions

The change in the structural order has a profound impact on electrochemical properties. In the materials studied in this work, the active metal, i.e., Fe, and the oxygen stoichiometry are the same, but the A-site metal is varied. The A-site metal does not directly participate in the electrochemical processes. However, the variation in the ionic radius of the A-site metal leads to a structural change, which has a major impact on electrochemical properties, where Ba_2_Fe_2_O_5_ shows a significantly enhanced activity for both OER and HER, compared to Sr_2_Fe_2_O_5_. Structural changes also lead to the improvement of pseudocapacitive properties of Ba_2_Fe_2_O_5_, which shows a considerably higher specific capacitance and energy density compared to Sr_2_Fe_2_O_5_. This study highlights the importance of structural order in determining the functional properties of oxides that are derived from the perovskite structure.

## Data Availability

Data will be made available by corresponding author upon reasonable request.

## Supplementary Materials

The following supporting information can be downloaded at: https://www.mdpi.com/article/10.3390/molecules28165947/s1, Figure S1: Rietveld refinement profiles using powder X-ray diffraction data for (a) Sr_2_Fe_2_O_5_ and (b) Ba_2_Fe_2_O_5_. Black cross symbols, red solid curve, green vertical tick marks, and the pink curve correspond to the experimental data, calculated model, Bragg peak positions, and the difference plot, respectively; Figure S2: Cyclic voltammograms for both compounds in the non-faradic region to obtain double layer capacitance (C_dl_) as shown in Figure 4b; Figure S3: Calibration of the Hg/HgO reference electrode in 1 M KOH, giving 0.923 V, which is nearly identical to that expected for 1 M KOH (pH = 14), i.e., 0.924 V. According to the Nernst equation, E_RHE_ = E_Hg/HgO_ + 0.059 pH + E⁰_Hg/HgO_, where E⁰_Hg/HgO_ = 0.098 V. Therefore, In 1 M KOH, E_RHE_ = E_Hg/HgO_ + 0.924 V. See the Experimental section for more information on calibration; Table S1: The refined structural parameters of Sr_2_Fe_2_O_5_ using PXRD data. Space group: *Ibm2*, a = 5.6750(1) Å, b = 15.5870(2) Å, c = 5.53261(1), R_p_ = 0.024, wR_p_ = 0.033, χ^2^ = 2.35%; Table S2: The refined structural parameters of Ba_2_Fe_2_O_5_ using PXRD data. Space group: *P2_1_/c,* a = 6.9750(1) Å, b = 11.7342(2) Å, c = 23.4503(3) Å, β = 98.7555(7)° R_p_ = 0.02, wR_p_ = 0.03, χ^2^ = 1.90%.
