# Supplementary material for: Enhancement of Electrocatalytic and Pseudocapacitive Properties as a Function of Structural Order in A2Fe2O5 (A = Sr, Ba)
Source: Molecules. 2023 Aug 8;28(16):5947. doi: 10.3390/molecules28165947 (PMC10459622; doi:10.3390/molecules28165947)
Supplement: Supplementary file 1 [file molecules-28-05947-s001.zip › molecules-2522907-supplementary.pdf]

Supporting Information

Enhancement of Electrocatalytic and Pseudocapacitive Properties as a Function of Structural Order in Perovskites $A_2Fe_2O_5$ (A = Sr, Ba)

Surendra B. Karki[a], Farshid Ramezanipour[a,*]

[a]Department of Chemistry, University of Louisville, Louisville, Kentucky 40292, USA
*Corresponding author. Email: farshid.ramezanipour@louisville.edu, Phone: +1(502) 852-7061
ORCID: 0000-0003-4176-1386

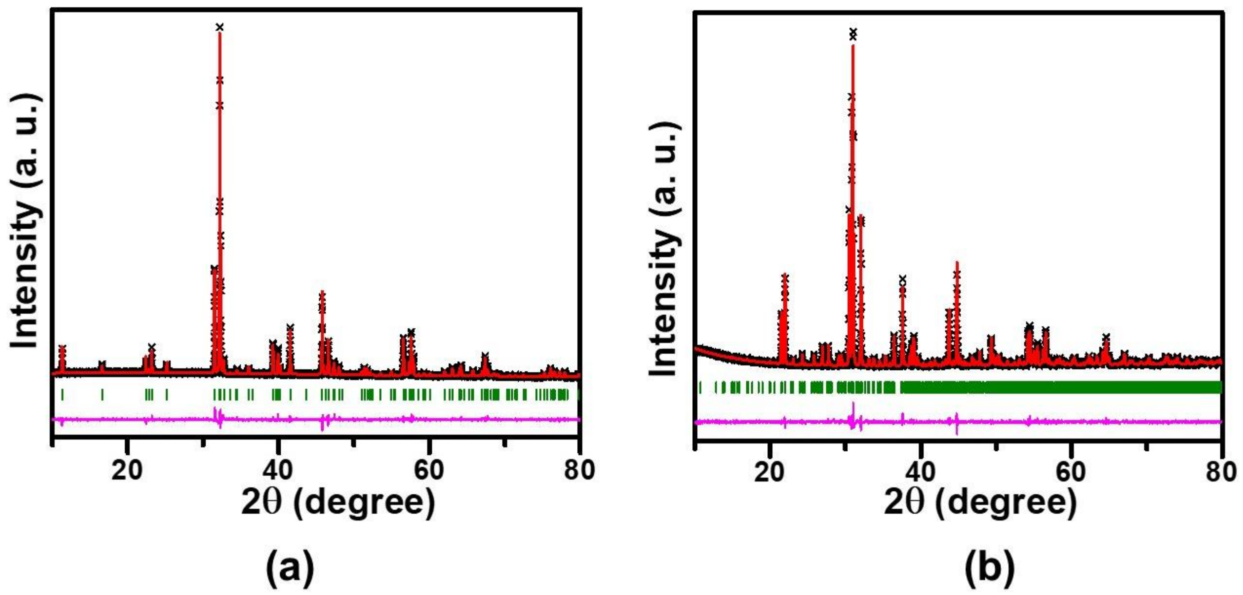

Figure S1. Rietveld refinement profiles using powder X-ray diffraction data for (a) $Sr_2Fe_2O_5$ and (b) $Ba_2Fe_2O_5$. Black cross symbols, red solid curve, green vertical tick marks, and the pink curve correspond to the experimental data, calculated model, Bragg peak positions, and the difference plot, respectively.

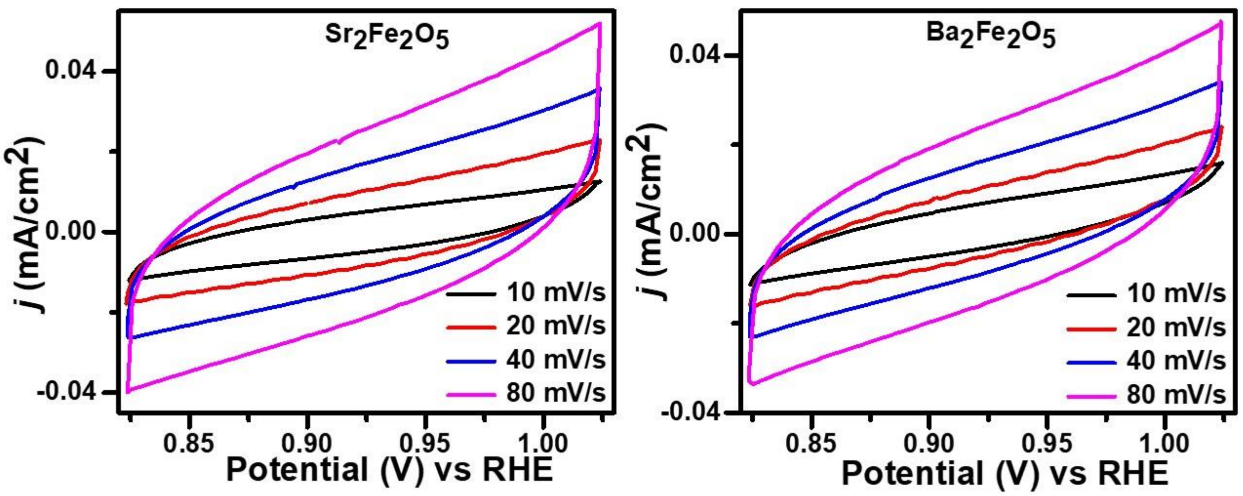

Figure S2. Cyclic voltammograms for both compounds in the non-faradic region to obtain double layer capacitance (C_{dl}) as shown in Figure 4b.

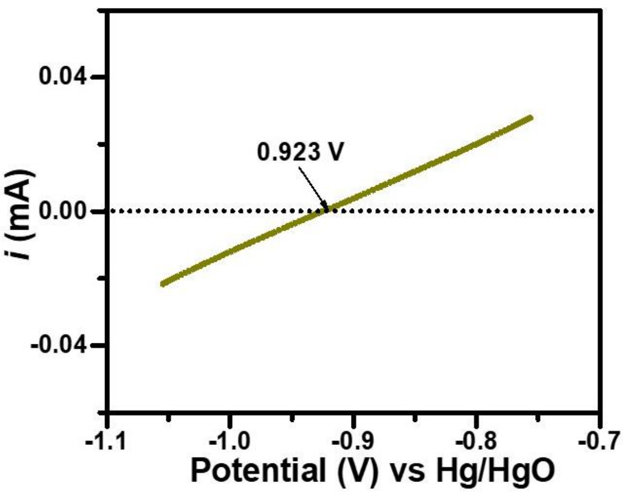

Figure S3. Calibration of the Hg/HgO reference electrode in 1 M KOH, giving 0.923 V, which is nearly identical to that expected for 1 M KOH (pH = 14), i.e., 0.924 V. According to the Nernst equation, $E_{RHE} = E_{Hg/HgO} + 0.059\ pH + E^{o}_{Hg/HgO}$, where $E^{o}_{Hg/HgO} = 0.098$ V. Therefore, In 1 M KOH, $E_{RHE} = E_{Hg/HgO} + 0.924$ V. See the Experimental section for more information on calibration.

Table S1: The refined structural parameters of $Sr_2Fe_2O_5$ using PXRD data. Space group: *Ibm2*, a = 5.6750(1) Å, b = 15.5870(2) Å, c = 5.53261(1), R_p = 0.024, wR_p = 0.033, χ^2 = 2.35%.

Element	*x*	*y*	*z*	Occupancy	Uiso	Multiplicity
Sr1	0.0167(5)	0.1104(1)	0.492(2)	1	0.016(1)	8
Fe1	0.00000	0.00000	-0.004(3)	1	0.021(2)	4
Fe2	0.934(1)	0.25000	0.954(2)	1	0.025(3)	4
O1	0.273(4)	0.9915(7)	0.266(5)	1	0.0250	8
O2	0.049(2)	0.1485(5)	0.034(3)	1	0.0250	8
O3	0.838(3)	0.250	0.575(4)	1	0.0250	4

Table S2: The refined structural parameters of $Ba_2Fe_2O_5$ using PXRD data. Space group: *P2₁/c*, a = 6.9750(1) Å, b = 11.7342(2) Å, c = 23.4503(3) Å, β = 98.7555(7)° R_p = 0.02, wR_p = 0.03, χ^2 = 1.90%.

Element	*x*	*y*	*z*	Occupancy	Uiso	Multiplicity
Ba1	0.053(1)	0.355(1)	0.1143(5)	1	0.016(4)	4
Ba2	0.251(2)	0.612(1)	0.3287(6)	1	0.032(5)	4
Ba3	0.133(1)	0.134(1)	0.3217(5)	1	0.019(4)	4
Ba4	0.321(2)	0.632(1)	0.0394(6)	1	0.033(4)	4
Ba5	0.053(2)	0.616(1)	0.6014(6)	1	0.013(4)	4
Ba6	0.556(2)	0.352(1)	0.2572(5)	1	0.037(5)	4
Ba7	0.354(2)	0.110(1)	0.0362(7)	1	0.021(4)	4
Fe1	0.529(4)	0.362(3)	0.100(1)	1	0.03(1)	4
Fe2	0.390(3)	0.584(2)	0.187(1)	1	0.01(9)	4
Fe3	0.082(4)	0.395(3)	0.259(2)	1	0.06(1)	4
Fe4	0.171(5)	0.156(2)	0.470(2)	1	0.02(1)	4
Fe5	0.420(4)	0.384(3)	0.398(1)	1	0.01(9)	4
Fe6	0.269(4)	0.102(2)	0.176(1)	1	0.01(1)	4
Fe7	0.145(5)	0.620(3)	0.466(2)	1	0.029(8)	4
O1	0	0	0	1	0.035	2
O2	0.29(1)	0.711(8)	0.148(4)	1	0.035	4
O3	-0.05(1)	0.287(8)	0.234(4)	1	0.035	4
O4	0.06(1)	0.041(7)	0.430(4)	1	0.035	4
O5	0.00(1)	0.247(9)	-0.002(4)	1	0.035	4
O6	0.27(1)	0.479(8)	0.431(4)	1	0.035	4
O7	0.70(1)	0.261(9)	0.073(5)	1	0.035	4
O8	0.05(1)	0.120(8)	0.111(5)	1	0.035	4
O9	0.42(1)	-0.030(7)	0.137(4)	1	0.035	4
O10	0.37(1)	0.260(7)	0.153(4)	1	0.035	4
O11	0.12(1)	-0.007(8)	0.207(5)	1	0.035	4
O12	0.46(1)	0.113(8)	0.251(4)	1	0.035	4
O13	0.17(1)	0.494(8)	0.209(4)	1	0.035	4
O14	0.57(1)	0.482(7)	0.148(4)	1	0.035	4
O15	0.29(1)	0.24(1)	0.422(5)	1	0.035	4
O16	0.38(1)	0.392(6)	0.030(5)	1	0.035	4
O17	0.25(1)	0.365(7)	0.317(5)	1	0.035	4
O18	0.62(1)	0.358(7)	0.466(5)	1	0.035	4
